# Trajectories and predictors of vicarious traumatization in Chinese college students during the COVID-19 pandemic: A longitudinal study

**DOI:** 10.3389/fpsyt.2022.1026905

**Published:** 2022-10-21

**Authors:** Hong Luo, Zhen Yu, Ju Li, Yujie Wang, Xiaopan Shi, Dan Luo, Jie Chen, Bing Xiang Yang

**Affiliations:** ^1^School of Basic Medicine, Hubei University of Arts and Science, Xiangyang, Hubei, China; ^2^School of Nursing, Wuhan University, Wuhan, Hubei, China; ^3^Department of Pain and Translational Symptom Science, University of Maryland School of Nursing, Baltimore, MD, United States

**Keywords:** COVID-19, vicarious traumatization, college students, trajectories, longitudinal study

## Abstract

**Objectives:**

This longitudinal study aimed to identify the trajectories and the predictors among sociodemographic and psychosocial variables at baseline of vicarious traumatization (VT) in Chinese college students during the COVID-19 pandemic.

**Materials and methods:**

A total of 544 Chinese college students enrolled in a public University in central China, majored in Clinical Medicine, Nursing, Musicology, Physics, etc., participated in this longitudinal study lasting 19 months. Three-wave (wave 1: February 2020; wave 2: November 2020; wave 3: September 2021) of data were collected. Resourcefulness Scale and the 10-item Kessler scale (K10) were only assessed in the first-wave survey, and the Event Scale-Revised (IES-R) was repeatedly measured in all three-wave surveys. A link to an online survey created by Questionnaire Star (https://www.wjx.cn/) was sent to the students to collect data. The Growth mixture modeling (GMM) and multiple logistic regression were used to identify the trajectories of VT and predictors for the distinct trajectories.

**Results:**

The incidence of VT at each wave varied from 9.9% at wave 1, 4.0% at wave 2, to 2.6% at wave 3. Three trajectories of VT were the medium-level escalating group (3.0%), medium-level maintaining group (32.3%), and the low-level descending group (64.7%). Seniors (OR = 1.575, 95% CI: 1.059–2.341; OR = 1.161, 95% CI: 1.043–1.293) and those with poor mental health status (OR = 1.101, 95% CI: 1.030–1.177; OR = 1.083, 95% CI: 1.060–1.106) at baseline were more likely to be classified into the medium-level escalating group and medium-level maintaining group, respectively. Additionally, females (OR = 3.601, 95% CI: 1.311–9.887) were more likely to be included in the medium-level escalating group.

**Conclusion:**

Targeted psychological interventions are urgently needed for students vulnerable to VT. Further studies with more representative samples, longer period of follow-up, and predictors based on scientific theoretical framework, are needed to update the findings.

## Introduction

The pandemic of Coronavirus disease 2019 (COVID-19) not only has far-ranging, profound, and adverse consequences on general physical and mental well-being but also sweeping changes in individuals’ daily activities worldwide for almost 3 years (1). College students enrolled before or during the pandemic were greatly affected including switching courses from in person to online, having difficulties in adjusting to the new academic activities, lacking peer support and emotional support, and lacking of autonomy (2, 3). Currently, a growing number of studies have identified college students as a vulnerable group more susceptible to having an array of psychological distress (e.g., anxiety symptoms, depressive symptomatology, and post-traumatic symptoms) as a result of the COVID-19 pandemic (4, 5). Notably, psychological distress seemed to be one of the most important causes for the decrease of empathy which is an important developmental skill associated with positive health outcomes and professional abilities (6). Psychological distress in college students during COVID-19 pandemic was likely promoted by the distance education (3), cognitive thinking style, the length of home confinement (2), vicarious traumatization (7), which in turn was significantly correlated with negative effects on daily life (e.g., sleep disturbances and loss of energy) and academic activities (e.g., difficulties in concentration and academic progress) (8), and negatively affected their health and study outcomes.

Vicarious traumatization (VT) occurred as a result of excessive empathy, initially referred to the symptoms of intrusion, avoidance, and arousal in professional psychotherapists who were indirectly affected by the bidirectional interactions with patients (9). Nowadays, VT is defined as exposure to traumatic experiences of others (10), which has been extended to the general population and other destructive disasters (11). The concept of VT is similar to post traumatic stress disorder (PTSD), the main difference between them lied in the source of stress; that is, individuals with VT usually witness traumatic events of others, and the stress is relatively “indirect” (9). VT was commonly detected during the COVID-19 pandemic and should not be ignored due to the growing and mounting volume of information that both official and low scientific value (12). It was reported that 80.83% psychiatric inpatients and 100% psychotherapists experienced VT symptoms (13). It is noteworthy that VT was mainly originated from sympathy for traumatic events and had also been a common psychological response during the COVID-19 pandemic, and VT played a crucial role in the development of general psychological distress (14).

Specifically, with the symptoms of irritability, fatigue, and loss of appetite, etc., VT can negatively alter one’s worldviews and perspective on life, transform internal beliefs and cognitions, change psychological and emotional needs, decrease self-esteem and sense of belonging (15, 16). However, the majority of studies explored psychological distress (e.g., anxiety and depression) as a result of the COVID-19 pandemic; their research subjects focused on health professionals or the general population, the topic on VT related to COVID-19 in college students are scarce and limited. Variables such as age, gender, the severity of the disaster, previous trauma, previous psychological diagnosis and life stress, and negative coping styles are associated with VT (17). Therefore, examining the influence and directly addressing these potential factors is crucial for the establishment and implementation of intervention strategies to reduce VT.

Notably, the change of VT is dynamic and developmental at different stages of the COVID-19 pandemic, which may also reveal heterogeneity in individuals. The growth mixture modeling (GMM), a person-centered approach, is flexible in identifying the heterogeneity and latent subgroups by using longitudinal data (18, 19). Moreover, previous evidence has shown that GMM yielded more precise parameter estimates than traditional analysis (20). Therefore, this longitudinal study aimed to explore and identify the trajectories and predictors of VT at three waves with a time window of 19 months in Chinese college students during the COVID-19 pandemic by GMM. Our study could offer insights into college students’ VT and promote specific psychological inventions for the targeted subgroups in college students during the COVID-19 pandemic.

## Materials and methods

### Participants

**FIGURE 1:**
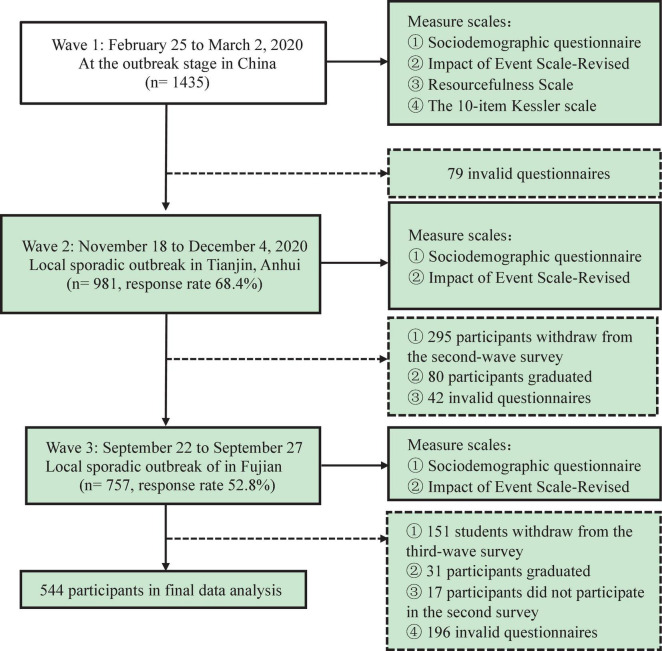
Flow diagram of time, background, scales, sample size of three-wave surveys.

This study was a 19 months follow-up longitudinal study, including three-wave surveys (wave 1: February 25 to March 2, 2020; wave 2: November 18 to December 4, 2020; wave 3: September 22 to September 27, 2021). Participants were recruited *via* a random sampling method from a public University in central China which located in Hubei province. The included participants could withdraw from the survey at any time. A mobile phone app-based questionnaire called Questionnaire Star^1^ was utilized to collect data (questionnaires and quick response codes presented in Supplementary Materials 1, 2). At wave 1, a total of 1,435 students accepted to participate in the survey, whereas the sample size dropped to 981 at wave 2, 757 participants completed the questionnaires at the end of wave 3, and 544 valid questionnaires from a sample of 1,435 were included in final data analysis (details presented in Figure 1).

The study protocol was approved by the Ethics Committee Hubei University of Arts and Science (Ethics Number: 2022-027). All the participants filled out electronic informed consent at the beginning of the questionnaire with the willingness to participate in this study.

### Measures

#### Sociodemographic questionnaire

This questionnaire includes participants’ gender, age, grade, major, residence location during the pandemic, and the severity of the COVID-19 epidemic in residence. Every participant was coded as an identification.

#### Vicarious traumatization measurement

Vicarious traumatization was measured by the Impact of Event Scale-Revised (IES-R), and was repeatedly assessed in three-wave surveys. This scale was originally developed by Horowitz, Wilner (21) and was widely used to evaluate post-traumatic symptoms (13) and VT. The IES-R in current studies is composed of three dimensions (e.g., intrusion, avoidance, and hyperarousal) with 22 items, and it is a Likert five-point scale ranging from 0 (no) to 4 (always). The cutoff point of 35 is used to classify the samples into non-VT (less than 35) and VT (≥35) groups (22). IES-R has been validated and verified in Chinese with good reliability and validity. The Cronbach’s alphas is 0.96 for the total scale, 0.93, 0.89, and 0.84 for the subscales (22).

#### Psychosocial variables associated with vicarious traumatization trajectories

##### Resourcefulness scale

The Resourcefulness Scale was first developed by Zauszniewski, Lai (23) and was used to assess the levels of resourcefulness. This scale consists of 28 items that belong to the subscales of personal resourcefulness (e.g., think about reward, think positively, keep busy, etc.) and social resourcefulness (e.g., listen to others, get help from others, talk with others, etc.), respectively. Each item is rated from 0 (never) to 4 (always). The Cronbach’s alphas of the validated Chinese Resourcefulness Scale is 0.852, and the content validity index (CVI) of the scale is 0.90 (24).

##### The 10-item Kessler scale (K10)

The 10-item Kessler scale is a widely used screening tool for non-specific psychological distress, which consists of 10 items (e.g., tired, nervous, hopeless, etc.) to elicit the frequency of depressive and anxiety symptoms over the past month with a Likert 4-point scale of frequency (25). The Cronbach’s alphas of the Chinese version of K10 is 0.80 (26), and this scale is used to assess the mental health status of college students at baseline in the first-wave survey in our study.

### Statistical analysis

To identify distinct trajectories of VT in Chinese college students, Mplus software (version 8.4) was adopted for GMM analysis. The fit indices for the accuracy and optimal model were Akaike Information Criterion (AIC), Bayesian Information Criterion (BIC), adjusted BIC (aBIC), Entropy value, Lo-Mendell-Rubin Likelihood ratio test (LMR), and Bootstrapped Likelihood ratio test (BLRT) (27, 28). The smaller values of AIC, BIC, and aBIC represent a better fitting effect for the model. The value of Entropy ranges from 0 to 1, and the larger value presents a more precise fitting model. Previous studies have reported that if the value of Entropy is ≥0.8, the accuracy of the model is preferred and will be as high as 90% (27, 29). Significant *P*-values of LMR and BLRT suggest that the category of the k-class model is better than that of k-1. For the predictors of VT trajectories, potential variables of gender, grade, age, residence location during the pandemic, the severity of epidemic in residence, resourcefulness, and mental health status at baseline were considered and performed by univariate tests. Variables with significant differences in univariate tests were included in the final multiple logistic regression, which was performed by SPSS software (version 22.0).

## Results

### Sociodemographic characteristics of the participants at baseline

The proportion of females (80.1%) was significantly higher than that of males (19.9%) among 544 students included in analysis who participated in three waves. Majors of the participants were enrolled in Clinical Medicine, Nursing, Musicology, Physics, English, Mathematics, etc. More than 50% of the participants were freshmen (52.8%), followed by sophomores (34.0%). Most participants (65.1%) were from Hubei province, 48.9% were from rural areas, 56.8% self-reported the epidemic of COVID-19 at wave 1 in their residence was not serious, 26.5% of them reported serious (Supplementary Table 1).

### Rates of vicarious traumatization at three waves

**FIGURE 2:**
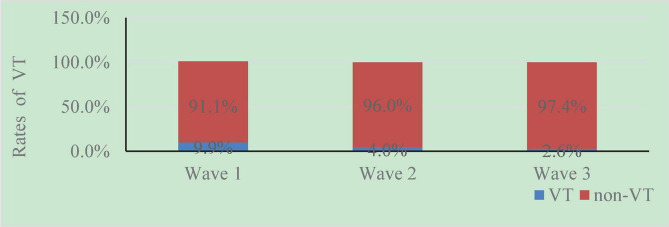
The rates of VT at different time points in college students.

As Figure 2 illustrated, the rate of VT has fallen dramatically across the COVID-19 pandemic. That is, 9.9% (54/544) of the participants self-reported having VT symptoms at wave 1, whereas the rates of students’ VT in later two-wave surveys were 4.0% (22/544) and 2.6% (14/544), respectively.

**FIGURE 3:**
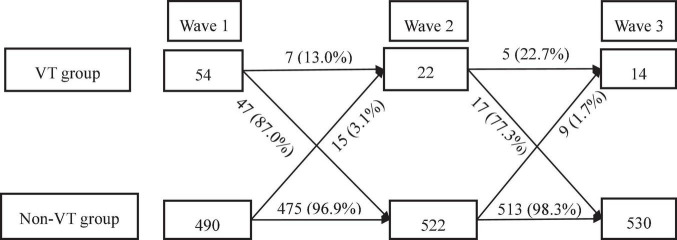
Flow chart of the transformation of VT and non-VT in three-wave surveys.

As Figure 3 presented, seven out of 54 students who were classified into the VT group still had symptoms of VT in the second-wave survey, and the other 47 students changed to the non-VT group; By contrast, fifteen students from the non-VT group transited to the VT group in the second-wave survey. In the third-wave survey, five out of 22 students remained in the VT group, and the other seventeen students changed to the non-VT group, whereas nine from 522 students in the non-VT group changed to the VT group.

### Trajectories of vicarious traumatization

To better describe the trajectories of VT among the 544 participants filled surveys at all three data collection time points, the fitting model indices of VT for the GMM approach was presented in Table 1. The three-class model yielded significant *P*-values for both LMR and BLRT, as well as the Entropy value (0.817) was higher than that of the two-class model (0.732). Additionally, the scree plot of aBIC value also indicated that the three-class model was reasonable (Supplementary Figure 1), and the attribution probability of the three trajectories of VT belonging to its category were between 0.854 and 0.938 (Supplementary Table 2). Therefore, the three-class GMM VT model was concluded to be optimal.

**TABLE 1:** Fitting indices for the trajectories of VT (*n* = 544).

Classes	AIC	BIC	aBIC	Entropy	*P* (LMR)	*P* (BLRT)	Category probability
1-class	11723.999	11758.390	11732.995	—	—	—	1.00
2-class	11673.208	11720.496	11685.578	0.732	0.00	0.000	0.221/0.779
**3-class**	**11653.169**	**11713.355**	**11668.913**	**0.817**	**0.038**	**0.000**	**0.647/0.323/0.030**
4-class	11642.186	11715.268	11661.303	0.775	0.119	0.013	0.305/0.141/0.017/0.537
5-class	11637.560	11723.539	11660.051	0.755	0.481	0.000	0.0515/0.138/0.024/0.529/0.257

Highlighted in bold is the optimal model.

**FIGURE 4:**
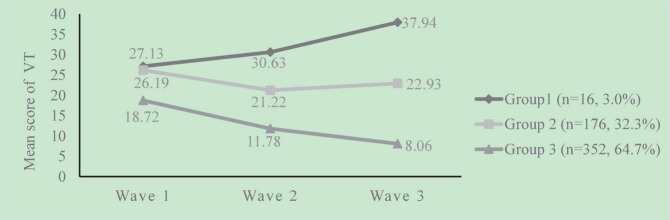
Trajectories of VT based on the three-class model. Group 1: “medium-level escalating group”; Group 2: “medium-level maintaining group”; Group 3: “low-level descending group.”

According to characteristics of the trajectories as Figure 4 illustrated, three distinct trajectories of VT were named “medium-level escalating group” (wave 1: mean = 27.13, SD = 10.29; wave 2: mean = 30.63, SD = 8.48; wave 3: mean = 37.94, SD = 5.00), “medium-level maintaining group” (wave 1: mean = 26.19, SD = 11.04; wave 2: mean = 21.22, SD = 8.86; wave 3: mean = 22.93, SD = 4.45), and “low-level descending group” (wave 1: mean = 18.72, SD = 9.23; wave 2: mean = 11.78, SD = 7.20; wave 3: mean = 8.06, SD = 4.46). The medium-level escalating group included sixteen (3.0%) participants, and the mean values of intercept (α) and slope (β) were 25.789 and 5.269 (*P* < 0.05), respectively. Participants in the medium-level escalating group reported a medium level VT in the first-wave survey, which showed a tendency of dramatic increasement over time. Especially, the mean score of VT was greater than the threshold of 35 in the third-wave survey. The medium-level maintaining group included 176 (32.3%) participants, and the mean values of intercept (α) and slope (β) were 24.366 and −1.30 (*P* < 0.01). Participants in the medium-level maintaining group had a medium level of VT in the first-wave survey, while the score gradually decreased over time, but still at medium-level (all means less than 30). The low-level descending group included 352 (64.7%) participants, and the mean values of intercept (α) and slope (β) were 17.884 and −4.978 (*P* < 0.05). Participants in the low-level descending group showed a relatively low level of VT in the first-wave survey and dramatically decreased across time.

### Predictors for the trajectories of vicarious traumatization

According to the results of univariate tests, variables of grade, residence location during the pandemic, the severity of the epidemic in residence, and level of resourcefulness at baseline were not the predictors for the trajectories of VT, whereas variables of gender, age, and mental health status at baseline were statistical significance (Supplementary Table 3), and were included in multiple logistic regression (Table 2). Taking the low-level descending group as reference, senior students (OR = 1.575, 95% CI: 1.059–2.341, *P* < 0.05; OR = 1.161, 95% CI: 1.043–1.293, *P* < 0.05) and those with poor mental health status (OR = 1.101, 95% CI: 1.030–1.177, *P* < 0.01; OR = 1.083, 95% CI: 1.060–1.106, *P* < 0.01) were more likely to be classified into the medium-level escalating group and medium-level maintaining group. Notably, females (OR = 3.601, 95% CI: 1.311–9.887, *P* < 0.01) were more likely to be included in the medium-level escalating group.

**TABLE 2:** Multiple logistic regression of predictors at baseline for the trajectories of VT.

Variables	Medium-level escalating group (*n* = 16)				Medium-level maintaining group (*n* = 176)			
	*B*	*SE*	OR	95% *CI*	*B*	*SE*	OR	95% *CI*
Age	0.454	0.202	1.575*	1.059∼2.341	0.150	0.055	1.161**	1.043∼1.293
Gender (female)	1.281	0.515	3.601**	1.311∼9.887	−0.007	0.162	0.993	0.723∼1.365
Mental health status	0.096	0.034	1.101**	1.030∼1.177	0.079	0.011	1.083**	1.060∼1.106

Taking the low-level descending group as reference.

*P < 0.05; **P < 0.01.

## Discussion

This longitudinal study consisted of three-wave surveys with 19 months of follow- up, reported the rates of VT at three waves, and identified three distinct dynamic trajectories of VT. In addition, a wide range of sociodemographic and psychosocial variables at baseline were added as possible predictors associated with the trajectories. These analyses allowed us to identify which groups were more vulnerable to VT, and establish specific psychological interventions to decrease the prevalence of VT related to COVID-19 in college students.

The three-wave surveys indicated that the prevalence of VT attached to COVID-19 in Chinese college students ranged from 9.9 to 2.6%, which revealed a significant effect of time on the change of VT. The severity and incidence of VT in Chinese college students in our study were significantly lower than that of in other populations (e.g., medical personnel, psychiatric inpatients, and the general public) (9, 13, 30). First, this notion may be because college students in Hubei province were under quarantine at home during the first-wave survey and had to spend considerable time and effort on online learning. Hence, they had limited time to gather information related to traumatic materials of COVID-19 through the internet. Second, the Chinese government adopted a series of effective measures which largely reduced the levels of psychological stress in college students, such as holding timely and transparent news conferences about the COVID-19 epidemic, ensuring the availability of essential commodities, and providing sufficient financial support, etc. Finally, it is notable that the heterogeneity in the measurement scales and differences in the time of the data collection for VT might be the potential reasons for different results.

Findings revealed a continuous descending of the point-prevalence estimates of the VT, and the rates were significantly different over three-wave surveys. These findings are similar to Amendola’s and Li’s, whose studies found significant reductions in anxiety in college students after the beginning of COVID-19 (31, 32). The potential reasons for the dramatic decrease in VT were as follows. First, in the last two-wave surveys, the confirmed case, infection rates, and mortality of COVID-19 decreased sharply in China. Although COVID-19 is an epidemic in some areas in China with un-expectations, the Chinese government always took timely and effective actions to address and control the further epidemic, which primarily established confidence among the general population. Second, isolation in social networks, difficulties in adapting to the online learning environment, and lack of social support during the lockdown at the first-wave survey, might result in higher VT levels in college students. While after lifting the lockdown, college students benefited a lot from face-to-face learning environment and gained more social support to reduce VT. Third, it was notable that there was no emerging confirmed case of COVID-19 during the last two-wave surveys in Hubei province.

The trajectory analysis provided dynamic information on VT overtime, allowed for heterogeneity and individual differences in trajectories and explained the characteristics of individual change in distinct potential groups. Our study identified three distinct VT trajectories: the medium-level escalating group, the medium-level maintaining group, and the low-level descending group. The majority of the participants were included in the low-level descending group. Students in this group had the lowest score of VT in the first-wave survey and dramatically decreased in later two-wave surveys. Whereas a small proportion of participants experienced the highest initial score and increased over time (medium-level escalating group), thus targeted psychological interventions should be established and adopted in this group. Notably, essential measures should also be taken on medium-level maintaining groups to change into an escalating group. Trauma-focused-cognitive behavior therapy (TF-CBT) was widely evidenced as a recommended psychological intervention for its both short-term and long-term efficacy on post-traumatic stress disorder (PTSD) in youth (33, 34), which may be helpful to build resilience and improve the individuals’ initial traumatized conditions. In addition, for the rapid development of digital health interventions and digital platforms, it is essential to incorporate psychological support for college students through listening and counseling services with the implementation of digital psychological interventions to contain, as far as possible, the evolution and structuring of psychopathological profiles during traumatic events (2).

Findings indicated that female gender, seniors, and poor mental health status of college students at baseline are more likely to be classified into the medium-level escalating group, highlighting that these subgroups are more vulnerable to VT. There is ample evidence of gender differences in psychological distress, and females are more likely to have higher levels (35–37). In addition, previous evidence has reported that females showed to be more active than males in neural networks associated with fear and arousal (38). Moreover, in our study, it is possible that the compassion and empathy for those traumatized lead to more disrupting effects on females. Similarly, studies showed that older children were more vulnerable to stress when exposed to war violence (39), and a larger proportion of students in senior high school experienced more severe psychological stress (40). Consistent with these findings, we found that the subgroup of senior students was more vulnerable to VT. Maybe, it is because the senior group in our study had more concern that the outbreak of COVID-19 could delay their academic career, negatively affect their job-hunting process, and exacerbate their financial situation. Additionally, Gao’s study reported that mental health is associated with general well-being (41), a protective factor for various psychological variables, such as resilience. Perhaps, college students with poor mental health are potentially susceptible to psychological distress, and actively engage in information-seeking to reduce uncertainty and anxiety during the COVID-19 pandemic (42), which in turn may increase the levels of VT.

### Limitations

Several limitations in this longitudinal study need to be addressed. First, participants included in this study were limited to one specific university in Hubei province, China. Second, this longitudinal study had only 19 months of follow-up. Hence, further studies should select more representative samples and conduct them with longer periods of follow-up. Third, predictors at baseline were not selected on the basis of a scientific and rigorous theoretical framework, and the effect of the interactions between the variables and the trajectory of VT is still unclear. Finally, this study exclusively depended on self-report scales but not diagnostic instruments, and an online survey is impossible to allow face-to-face in-depth interviews or direct observations.

### Implications

Our study revealed the changes of VT in Chinese college students and three distinct trajectories of VT, highlighted variables of gender, age, and mental health status were of vital importance. They might be of substantial interest to policy-makers and researchers to establish and implement specific psychological inventions for the targeted subgroups. Notably, students in the medium-level escalating group should be the most concerned, followed by the medium-level maintaining group. More potential variables based on a rigorous theoretical framework should be examined to update robust and comprehensive evidence on this topic. Studies that combine mixed research methods, such as qualitative and quantitative research, are urgently needed. Psychological stress and other risk factors, such as negative coping styles and resilience, should be taken seriously. Further studies could also develop interventions targeting these psychological factors to reduce VT.

## Conclusion

Findings indicated that the overall rate of COVID-19 related to VT in Chinese college students decreased dramatically across time. Three distinct trajectories of VT were identified, those in the medium-level escalating group were the most concerned, and the highest score at the third wave indicated targeted interventions are urgently needed as soon as possible. Essential interventions are also needed to establish and implement for students who are more vulnerable to VT, such as seniors, females, and students with poor mental health status. Further well-designed multicentric studies with more representative samples, longer period of follow-up longitudinal studies, and predictors with a scientific theoretical framework, are needed to update the outcomes of this study.

## Data availability statement

The original contributions presented in this study are included in the article/Supplementary material, further inquiries can be directed to the corresponding authors.

## Ethics statement

The studies involving human participants were reviewed and approved by Ethics Committee Hubei University of Arts and Science. The patients/participants provided their written informed consent to participate in this study.

## Author contributions

HL contributed to the work of conceptualization, methodology, data analysis, and wrote the original manuscript under the guidance of DL, JC, and BY. ZY was primarily responsible for conceptualization and data collection. JL, YW, and XS participated in the work of data collection. All authors approved the final manuscript.

## Footnotes

1
https://www.wjx.cn/
