# Supplementary material for: Trajectories and predictors of vicarious traumatization in Chinese college students during the COVID-19 pandemic: A longitudinal study
Source: Front Psychiatry. 2022 Oct 21;13:1026905. doi: 10.3389/fpsyt.2022.1026905 (PMC9633659; doi:10.3389/fpsyt.2022.1026905)
Supplement: Supplementary file 1 [file Data_Sheet_1.docx]

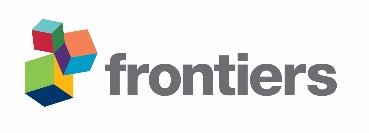


Supplementary Material 1

An Investigation on vicarious traumatization and its predictors in Chinese college students during the COVID-19 pandemic (Wave 1)

Dear students:

As we all know, China has experienced an outbreak of Coronavirus disease 2019 (COVID-19) since December 2019. The epidemic of COVID-19 has a wide range of prevalence, strong infectivity, no obvious symptoms in the incubation period, and its had a huge threat and impact on individuals’ health, psychology, cognition, and behavior.

A growing number of studies have identified college students as a vulnerable group more susceptible to having an array of psychological distress. Therefore, this study aimed to identify the trajectories and predictors of vicarious traumatization in Chinese college students during the COVID-19 pandemic, and offer insights into college students’ vicarious traumatization and promote specific psychological inventions for the targeted subgroups in college students during the COVID-19 pandemic.

If you are willing to participate in the three-wave investigations, please answer the following items truthfully and carefully according to your psychological experience, and you can withdraw from the survey at any time. All your information is only for research and will not be disclosed. Your true and accurate answers are crucial to our research. Thanks again for your support.

If you are willing to participate in this research, please sign your name in the signature area. Thank you for your cooperation.

Full name:

Specific date:

Please select from the following items or fill in your general information.

1. Your gender:

○ Male

○ Female

2. Majors:

3. Grade:

○ Freshman

○ Sophomore

○ Junior

○ Senior

4. Age:

5. Where you live:

○ City

○ Town

○ Rural area

6. Residence location during the pandemic

○ Urban areas

○ Sub-urban areas

○ Rural areas

7. Severity of epidemic in the residence

○ No epidemic

○ Not serious

○ Serious

○ Extreme serious

In your daily life, you have different decision-making styles. Please choose the best option from the following items.

1. When you are doing a boring job, you will think about the interesting part of it, and also think about the benefits when the work is completed.

○ Never ○ Rarely ○ Sometimes ○ Often  ○ Always

2. When you have to do something that makes you nervous, you try to imagine how to overcome it.

○ Never ○ Rarely ○ Sometimes ○ Often ○ Always

3. As long as you change your mind on something, your feelings will usually change with it.

○ Never ○ Rarely ○ Sometimes ○ Often ○ Always

4. When you feel depressed, you will try to think of something pleasant.

○ Never ○ Rarely ○ Sometimes ○ Often ○ Always

5. When you face a problem, you will try to find a solution to it.

○ Never ○ Rarely ○ Sometimes ○ Often ○ Always

6. When some unpleasant thoughts bother you, you will try to think of something pleasant.

○ Never ○ Rarely ○ Sometimes ○ Often ○ Always

7. When you are depressed, you will do something you like to keep busy.

○ Never ○ Rarely ○ Sometimes ○ Often ○ Always

8. When you are difficult to make a decision, you will find ways to calm yourself down.

○ Never ○ Rarely ○ Sometimes ○ Often ○ Always

9. To overcome the frustration caused by failure, you often tell yourself that things are not so tragic, and you can still make some efforts for it.

○ Never ○ Rarely ○ Sometimes ○ Often ○ Always

10. When you feel that you are eager for quick success and instant benefit, you will tell yourself to stop, think about it and then act.

○ Never ○ Rarely ○ Sometimes ○ Often ○ Always

11. Even if you are very angry with someone, you will still watch your actions carefully.

○ Never ○ Rarely ○ Sometimes ○ Often ○ Always

12. When you need to make a decision, you will first find out all the options and not make a decision too quickly.

○ Never ○ Rarely ○ Sometimes ○ Often ○ Always

13. When you find that you are bound to be late for some important meetings or appointments, you will tell yourself to keep calm.

○ Never ○ Rarely ○ Sometimes ○ Often ○ Always

14. When you have a lot of things to do, you usually plan well.

○ Never ○ Rarely ○ Sometimes ○ Often ○ Always

15. When you are short of money, you will record all your expenses, so that you can be more careful in arranging your budget in the future.

○ Never ○ Rarely ○ Sometimes ○ Often ○ Always

16. When you find it difficult to focus on a certain task, you will divide it into smaller parts and implement it in sections.

○ Never ○ Rarely ○ Sometimes ○ Often ○ Always

17. When you feel sad, talking to someone will help you.

○ Never ○ Rarely ○ Sometimes ○ Often ○ Always

18. When you find it difficult to make a decision, you will ask others to think for you.

○ Never ○ Rarely ○ Sometimes ○ Often ○ Always

19. When you feel pain and discomfort, you will ask for help from the doctors, nurses or professional staffs.

○ Never ○ Rarely ○ Sometimes ○ Often ○ Always

20. When you feel confused, you will rely on others to help you.

○ Never ○ Rarely ○ Sometimes ○ Often ○ Always

21. With regard to your health, you will follow and implement the advice given to you by

others.

○ Never ○ Rarely ○ Sometimes ○ Often ○ Always

22. When you have many things to do, you will ask others to help you.

○ Never ○ Rarely ○ Sometimes ○ Often ○ Always

23. If you don't have enough money to pay the bill, you will borrow money from someone.

○ Never ○ Rarely ○ Sometimes ○ Often ○ Always

24. When you are angry, you will talk with others to vent your feelings.

○ Never ○ Rarely ○ Sometimes ○ Often ○ Always

25. When you encounter a situation you have not faced in the past, you will turn to someone who has faced the same situation.

○ Never ○ Rarely ○ Sometimes ○ Often ○ Always

26. When you have to do something you don't like, you will ask others how they did it.

○ Never ○ Rarely ○ Sometimes ○ Often ○ Always

27. If you need to go somewhere, you will ask someone to take you there.

○ Never ○ Rarely ○ Sometimes ○ Often ○ Always

28. When you are in listlessness, you will be with others and get more energy from their relationship.

○ Never ○ Rarely ○ Sometimes ○ Often ○ Always

Do you have the following psychological problems in the past four weeks during the COVID-19 pandemic? If you have, please choose the best option.

1. You often feel tired for no reason.

○ All the time ○ Most of the time  ○ Sometimes ○ On occasion ○ No time

2. You feel nervous.

○ All the time ○ Most of the time ○ Sometimes ○ On occasion ○ No time

3. You are so nervous that nothing can calm you down.

○ All the time ○ Most of the time ○ Sometimes ○ On occasion ○ No time

4. You feel helpless.

○ All the time ○ Most of the time ○ Sometimes ○ On occasion ○ No time

5. You feel insomnia and uneasy.

○ All the time ○ Most of the time ○ Sometimes ○ On occasion ○ No time

6. You are on tenterhooks.

○ All the time ○ Most of the time ○ Sometimes ○ On occasion ○ No time

7. You feel depressed.

○ All the time ○ Most of the time ○ Sometimes ○ On occasion ○ No time

8. You find it difficult to do anything.

○ All the time ○ Most of the time ○ Sometimes ○ On occasion ○ No time

9. You feel that nothing can arouse your interest.

○ All the time ○ Most of the time ○ Sometimes ○ On occasion ○ No time

10. You feel worthless.

○ All the time ○ Most of the time ○ Sometimes ○ On occasion ○ No time

Do you have the following psychological problems in the past four weeks during the COVID-19 pandemic? If you have, please choose the best option.

1. You will be touched by anything that related to the COVID-19.

○ No time ○ Seldom ○ Sometimes  ○ Often ○ Always

2. It is difficult for you to sleep safely until dawn.

○ No time ○ Seldom ○ Sometimes ○ Often ○ Always

3. Other things will remind you of the COVID-19.

○ No time ○ Seldom ○ Sometimes ○ Often ○ Always

4. Even if you don't want to think about the COVID-19, you will still think about it.

○ No time ○ Seldom ○ Sometimes ○ Often ○ Always

5. Something about the COVID-19 will suddenly appear in your mind.

○ No time ○ Seldom ○ Sometimes ○ Often ○ Always

6. Your behaviors and feelings are often affected by the COVID-19.

○ No time ○ Seldom ○ Sometimes ○ Often ○ Always

7. You have strong mood swings due to the COVID-19.

○ No time ○ Seldom ○ Sometimes ○ Often ○ Always

8. You dream about the scene related to the COVID-19.

○ No time ○ Seldom ○ Sometimes ○ Often ○ Always

9. Whenever you think of the COVID-19 or other things that remind you of it, you will try your best not to upset yourself.

○ No time ○ Seldom ○ Sometimes ○ Often ○ Always

10. It seems that the COVID-19 has never happened or is not true.

○ No time ○ Seldom ○ Sometimes ○ Often ○ Always

11. You try to stay away from everything that reminds you of the COVID-19.

○ No time ○ Seldom ○ Sometimes ○ Often ○ Always

12. You don't think about the COVID-19.

○ No time ○ Seldom ○ Sometimes ○ Often ○ Always

13. You realize that you still have a lot of thoughts about the COVID-19, but you haven't dealt with them.

○ No time ○ Seldom ○ Sometimes ○ Often ○ Always

14. You feel a little numb about the COVID-19.

○ No time ○ Seldom ○ Sometimes ○ Often ○ Always

15. You want to forget about the COVID-19.

○ No time ○ Seldom ○ Sometimes ○ Often ○ Always

16. You try your best not to mention about the COVID-19.

○ No time ○ Seldom ○ Sometimes ○ Often ○ Always

17. You are irritable.

○ No time ○ Seldom ○ Sometimes ○ Often ○ Always

18. You feel nervous and easily frightened.

○ No time ○ Seldom ○ Sometimes ○ Often ○ Always

19. You can't sleep.

○ No time ○ Seldom ○ Sometimes ○ Often ○ Always

20. You find it is difficult for you to focus attention.

○ No time ○ Seldom ○ Sometimes ○ Often ○ Always

21. When you think of the COVID-19, it will lead to negative reactions on your body, such as sweating, panic, frequent urination, etc.

○ No time ○ Seldom ○ Sometimes ○ Often ○ Always

22. You feel alert.

○ No time ○ Seldom ○ Sometimes ○ Often ○ Always

An Investigation on vicarious traumatization and its predictors in Chinese college students during the COVID-19 pandemic (Wave 2)

Do you have the following psychological problems in the past four weeks during the COVID-19 pandemic? If you have, please choose the best option.

1. You will be touched by anything that related to the COVID-19.

○ No time ○ Seldom ○ Sometimes ○ Often ○ Always

2. It is difficult for you to sleep safely until dawn.

○ No time ○ Seldom ○ Sometimes ○ Often ○ Always

3. Other things will remind you of the COVID-19.

○ No time ○ Seldom ○ Sometimes ○ Often ○ Always

4. Even if you don't want to think about the COVID-19, you will still think about it.

○ No time ○ Seldom ○ Sometimes ○ Often ○ Always

5. Something about the COVID-19 will suddenly appear in your mind.

○ No time ○ Seldom ○ Sometimes ○ Often ○ Always

6. Your behaviors and feelings are often affected by the COVID-19.

○ No time ○ Seldom ○ Sometimes ○ Often ○ Always

7. You have strong mood swings due to the COVID-19.

○ No time ○ Seldom ○ Sometimes ○ Often ○ Always

8. You dream about the scene related to the COVID-19.

○ No time ○ Seldom ○ Sometimes ○ Often ○ Always

9. Whenever you think of the COVID-19 or other things that remind you of it, you will try your best not to upset yourself.

○ No time ○ Seldom ○ Sometimes ○ Often ○ Always

10. It seems that the COVID-19 has never happened or is not true.

○ No time ○ Seldom ○ Sometimes ○ Often ○ Always

11. You try to stay away from everything that reminds you of the COVID-19.

○ No time ○ Seldom ○ Sometimes ○ Often ○ Always

12. You don't think about the COVID-19.

○ No time ○ Seldom ○ Sometimes ○ Often ○ Always

13. You realize that you still have a lot of thoughts about the COVID-19, but you haven't dealt with them.

○ No time ○ Seldom ○ Sometimes ○ Often ○ Always

14. You feel a little numb about the COVID-19.

○ No time ○ Seldom ○ Sometimes ○ Often ○ Always

15. You want to forget about the COVID-19.

○ No time ○ Seldom ○ Sometimes ○ Often ○ Always

16. You try your best not to mention about the COVID-19.

○ No time ○ Seldom ○ Sometimes ○ Often ○ Always

17. You are irritable.

○ No time ○ Seldom ○ Sometimes ○ Often ○ Always

18. You feel nervous and easily frightened.

○ No time ○ Seldom ○ Sometimes ○ Often ○ Always

19. You can't sleep.

○ No time ○ Seldom ○ Sometimes ○ Often ○ Always

20. You find it is difficult for you to focus attention.

○ No time ○ Seldom ○ Sometimes ○ Often ○ Always

21. When you think of the COVID-19, it will lead to negative reactions on your body, such as sweating, panic, frequent urination, etc.

○ No time ○ Seldom ○ Sometimes ○ Often ○ Always

22. You feel alert.

○ No time ○ Seldom ○ Sometimes ○ Often ○ Always

An Investigation on vicarious traumatization and its predictors in Chinese college students during the COVID-19 pandemic (Wave 3)

Do you have the following psychological problems in the past four weeks during the COVID-19 pandemic? If you have, please choose the best option.

1. You will be touched by anything that related to the COVID-19.

○ No time ○ Seldom ○ Sometimes ○ Often ○ Always

2. It is difficult for you to sleep safely until dawn.

○ No time ○ Seldom ○ Sometimes ○ Often ○ Always

3. Other things will remind you of the COVID-19.

○ No time ○ Seldom ○ Sometimes ○ Often ○ Always

4. Even if you don't want to think about the COVID-19, you will still think about it.

○ No time ○ Seldom ○ Sometimes ○ Often ○ Always

5. Something about the COVID-19 will suddenly appear in your mind.

○ No time ○ Seldom ○ Sometimes ○ Often ○ Always

6. Your behaviors and feelings are often affected by the COVID-19.

○ No time ○ Seldom ○ Sometimes ○ Often ○ Always

7. You have strong mood swings due to the COVID-19.

○ No time ○ Seldom ○ Sometimes ○ Often ○ Always

8. You dream about the scene related to the COVID-19.

○ No time ○ Seldom ○ Sometimes ○ Often ○ Always

9. Whenever you think of the COVID-19 or other things that remind you of it, you will try your best not to upset yourself.

○ No time ○ Seldom ○ Sometimes ○ Often ○ Always

10. It seems that the COVID-19 has never happened or is not true.

○ No time ○ Seldom ○ Sometimes ○ Often ○ Always

11. You try to stay away from everything that reminds you of the COVID-19.

○ No time ○ Seldom ○ Sometimes ○ Often ○ Always

12. You don't think about the COVID-19.

○ No time ○ Seldom ○ Sometimes ○ Often ○ Always

13. You realize that you still have a lot of thoughts about the COVID-19, but you haven't dealt with them.

○ No time ○ Seldom ○ Sometimes ○ Often ○ Always

14. You feel a little numb about the COVID-19.

○ No time ○ Seldom ○ Sometimes ○ Often ○ Always

15. You want to forget about the COVID-19.

○ No time ○ Seldom ○ Sometimes ○ Often ○ Always

16. You try your best not to mention about the COVID-19.

○ No time ○ Seldom ○ Sometimes ○ Often ○ Always

17. You are irritable.

○ No time ○ Seldom ○ Sometimes ○ Often ○ Always

18. You feel nervous and easily frightened.

○ No time ○ Seldom ○ Sometimes ○ Often ○ Always

19. You can't sleep.

○ No time ○ Seldom ○ Sometimes ○ Often ○ Always

20. You find it is difficult for you to focus attention.

○ No time ○ Seldom ○ Sometimes ○ Often ○ Always

21. When you think of the COVID-19, it will lead to negative reactions on your body, such as sweating, panic, frequent urination, etc.

○ No time ○ Seldom ○ Sometimes ○ Often ○ Always

22. You feel alert.

○ No time ○ Seldom ○ Sometimes ○ Often ○ Always

***Supplementary Material 2***

In China, WeChat is one of the most popular apps, and Wenjuanxin is a frequently-used data collection tool. Specifically, participants were informed the background, purpose, and informed consent of the study at the beginning of investigations. Afterwards, individuals who is voluntary to participate in the study will scan a uniform QR code to finish a questionnaire as depicted in Supplementary Material 1 via Wechat app or directly click on the website. The three waves of QR codes and websites are as follows:

| 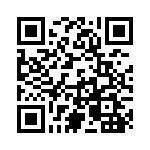 https://www.wjx.cn/vj/mbDXlkO.aspx | QR code of wave 1  (Wave 1: February 25 to March 2, 2020) |
| --- | --- |
| **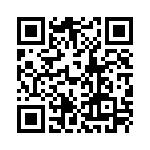** https://www.wjx.cn/jq/96292016.aspx | QR code of wave 2  (Wave 2: November 18 to December 4, 2020) |
| **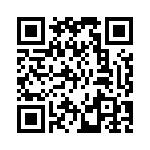** https://www.wjx.cn/vj/mbDXlkO.aspx | QR code of wave 3  (Wave 3: September 22 to September 27, 2021) |

# Supplementary Figures and Tables

**2.1 Supplementary Figures**

Supplementary Figure 1: Scree plot of aBIC value

- 1. **Supplementary Tables**

Supplementary Table 1: Demographic characteristics of the participants at baseline (n, %)

| Variables | n/$\bar{x}$ | Percentage (%)/sd |
| --- | --- | --- |
| Gender |  |  |
| Male | 108 | 19.9% |
| Female | 436 | 80.1% |
| Grade |  |  |
| Freshman | 287 | 52.8% |
| Sophomore | 185 | 34.0% |
| Junior | 67 | 12.3% |
| Senior | 5 | 0.9% |
| Residence location during the pandemic |  |  |
| Urban areas | 124 | 22.8% |
| Sub-urban areas | 154 | 28.3% |
| Rural areas | 266 | 48.9% |
| Severity of epidemic in residence |  |  |
| No epidemic | 79 | 14.5% |
| Not serious | 309 | 56.8% |
| Serious | 144 | 26.5% |
| Extreme serious | 12 | 2.2% |

Supplementary Table 2 Trajectories of vicarious traumatization of each group belong to its category

|  | Group 1 | Group 2 | Group 3 |
| --- | --- | --- | --- |
| Group 1 | 0.854 | 0.146 | 0.000 |
| Group 2 | 0.024 | 0.888 | 0.088 |
| Group 3 | 0.000 | 0.062 | 0.938 |

Note: Group 1: medium-level escalating group; Group 2: medium-level maintaining group; Group 3: low-level descending group.

Supplementary Table 3: The predictors of the trajectory class of vicarious traumatization

| Variables | Group 1 | Group 2 | Group 3 | χ^2^*/Z* | *P* |
| --- | --- | --- | --- | --- | --- |
| Gender |  |  |  | -2.34 | **0.02^*^** |
| Male | 7 (43.8%) | 32 (18.2%) | 69 (19.6) |  |  |
| Female | 9 (56.3%) | 144 (81.8%) | 283 (80.4) |  |  |
| Grade |  |  |  | 5.60 | 0.06 |
| Freshman | 8 (50.0%) | 87 (49.4%) | 192 (54.5%) |  |  |
| Sophomore | 4 (25.0%) | 59 (33.5%) | 122 (34.7%) |  |  |
| Junior | 4 (25.0%) | 29 (16.5%) | 34 (9.7%) |  |  |
| Senior | 0 (0%) | 1(0.6%) | 4 (1.1%) |  |  |
| Age (years) | 19.94±1.34 | 19.51±1.22 | 19.34±1.11 | 8.24 | **0.02^*^** |
| Residence |  |  |  | 3.56 | 0.17 |
| Urban areas | 2 (12.5%) | 34 (19.3%) | 88 (25.0%) |  |  |
| Sub-urban areas | 4 (25.0%) | 54 (30.7%) | 96 (27.3%) |  |  |
| Rural areas | 10 (62.5%) | 88 (50.0%) | 168 (47.7%) |  |  |
| Severity of epidemic in residence |  |  |  | 4.88 | 0.09 |
| No epidemic | 3 (18.8%) | 22 (12.5%) | 54 (15.3%) |  |  |
| Not serious | 8 (50.0%) | 96 (54.5%) | 205 (58.2%) |  |  |
| Serious | 5 (31.3%) | 56 (31.8%) | 83 (23.6%) |  |  |
| Extreme serious | 0 (0%) | 2 (1.1%) | 10 (2.8%) |  |  |
| Resourcefulness score | 94.87±12.83 | 97.12±10.78 | 97.63±10.87 | 2.67 | 0.26 |
| Mental health status | 7.38±7.01 | 6.90±6.23 | 4.17±5.31 | 77.60 | **0.00^**^** |

Note: Group 1: medium-level escalating group, Group 2: medium-level maintaining group; Group 3: low-level descending group. **^**^** *P*＜0.01; **^*^** *P*＜0.05.
